# Low bone mineral density is associated with gray matter volume decrease in UK Biobank

**DOI:** 10.3389/fnagi.2023.1287304

**Published:** 2023-11-03

**Authors:** Polona Kalc, Robert Dahnke, Felix Hoffstaedter, Christian Gaser

**Affiliations:** ^1^Structural Brain Mapping Group, Department of Neurology, Jena University Hospital, Jena, Germany; ^2^Structural Brain Mapping Group, Department of Psychiatry and Psychotherapy, Jena University Hospital, Jena, Germany; ^3^Brain and Behaviour (INM-7), Institute of Neuroscience and Medicine, Forschungszentrum Jülich, Jülich, Germany; ^4^Institute of Systems Neuroscience, Medical Faculty, Heinrich Heine University Düsseldorf, Düsseldorf, Germany; ^5^German Center for Mental Health (DZPG), Jena-Halle-Magdeburg, Germany

**Keywords:** bone mineral density, osteoporosis, VBM, Alzheimer’s disease, UK Biobank

## Abstract

**Objectives:**

Previous research has found an association of low bone mineral density (BMD) and regional gray matter (GM) volume loss in Alzheimer’s disease (AD). We were interested whether BMD is associated with GM volume decrease in brains of a healthy elderly population from the UK Biobank.

**Materials and methods:**

T1-weighted images from 5,518 women (*M*_Age_ = 70.20, SD = 3.54; age range: 65–82 years) and 7,595 men (*M*_Age_ = 70.84, SD = 3.68; age range: 65–82 years) without neurological or psychiatric impairments were included in voxel-based morphometry (VBM) analysis in CAT12 with threshold-free-cluster-enhancement (TFCE) across the whole brain.

**Results:**

We found a significant decrease of GM volume in women in the superior frontal gyri, middle temporal gyri, fusiform gyri, temporal poles, cingulate gyri, precunei, right parahippocampal gyrus and right hippocampus, right ventral diencephalon, and right pre- and postcentral gyrus. Only small effects were found in men in subcallosal area, left basal forebrain and entorhinal area.

**Conclusion:**

BMD is associated with low GM volume in women but less in men in regions afflicted in the early-stages of AD even in a sample without neurodegenerative diseases.

## 1. Introduction

In the fields of neuroscience and neurology, the role of bone tissue on brain metabolism has long been neglected, however, novel insights in the bone-brain crosstalk are starting to pique interest of a wider research community. Burgeoning findings show that the bone is an important endocrine organ with intensive bidirectional communication with the brain (and other organs) (Guntur and Rosen, 2012; Rousseaud et al., 2016), is implicated in mood and cognition (Oury et al., 2013; Khrimian et al., 2017; Obri et al., 2018; Nakamura et al., 2021) as well as in multiple neurological, psychiatric and neurodevelopmental disorders (Kelly et al., 2020), such as Parkinson’s disease (Sleeman et al., 2016; Tassorelli et al., 2017), multiple sclerosis (Bisson et al., 2019), autism spectrum disorder (Neumeyer et al., 2015).

The connection between bone health (reflected by bone mineral density and/or bone mineral content, measured by dual-energy X-ray absorptiometry (DXA) or estimated by quantitative ultrasound) and cognition was intensively investigated in women during the 90’s and early 00’s. Bone mineral density was then identified as a surrogate marker of low estrogen levels in postmenopausal women (Tan et al., 2005). Results from these studies indicated a connection between low BMD and an increased risk of Alzheimer’s disease (AD) in women (Lui et al., 2003; Tan et al., 2005), and later also in men (Zhou et al., 2011). The role of BMD in AD was further considered by VBM studies that found an association between low BMD and a decreased gray matter volume in hypothalamic and limbic regions (Loskutova et al., 2010) and in a recent study from Takano et al. (2020), which showed an association between regional gray matter decline in left precuneus and BMD loss in male AD patients but not in women.

Given the consistent reports on the connection of bone measures and brain aging from the researchers associated with the UK Biobank research consortium (Miller et al., 2016; Smith et al., 2019), we wanted to test whether BMD is associated with gray matter volume changes also on a larger healthy sample from the UK Biobank.^1^

## 2. Methods

### 2.1. Participants

This research has been conducted using data from UK Biobank under the application number 41655. The UK Biobank is a biomedical database and research resource that contains genetic, lifestyle and health information from half a million UK Biobank participants (see text footnote 1). UKB holds the ethical approval from the North West Multi-Centre Research Ethics Committee (MREC) and is in possession of informed consents from the study cohort participants.

Due to a predominantly white sample in the UK Biobank and with regard to a possible effect of ethnic background on bone measures (Jorgetti et al., 2014; Zengin et al., 2015; Popp et al., 2017), the analyses were applied only to a sample of participants of British and Irish descent who underwent brain magnetic resonance (MR) imaging and had available BMD measures. Participants with pre-existing neurological or psychiatric diagnoses were excluded from the analyses. Further exclusion criteria were based on ICD-10 diagnosis of bone disorders and the use of medication that can affect bone metabolism (e.g., glucocorticoids, see Supplementary material for detailed exclusion criteria). We limited our analyses to a subset of subjects older than 65 years as men and women start to lose bone mass at a similar rate in this age range (National Institutes of Health Osteoporosis and Related Bone Diseases National Resource Center, 2023).

The analyses were conducted on a sample of 5,518 women (*M*_Age_ = 70.20, SD = 3.54; age range: 65–82 years) and 7,595 men (*M*_Age_ = 70.84, SD = 3.68; age range: 65–82 years).

### 2.2. Bone mineral density data acquisition

Bone mineral density measures of the left femoral neck were obtained by dual energy X-ray absorptiometry (DXA) scanning with iDXA instrument (GE-Lunar, Madison, WI). See https://biobank.ndph.ox.ac.uk/showcase/ukb/docs/DXA_explan_doc.pdf for a detailed report of the acquisition procedure. DXA scanning is a state-of-the-art assessment method for diagnosing osteoporosis (Arabi et al., 2007; Blake and Fogelman, 2007). A T-score instead of a raw score was used for femoral neck bone mineral density, representing a person’s relative bone stiffness of femoral neck in comparison to that of a healthy young adult of their sex.

### 2.3. MR data acquisition and processing

All the information regarding MR data acquisition is available in the UK Biobank Imaging documentation.^2^ In short, T1-weighted MR images were acquired on a Siemens Skyra 3T scanner with a 32-channel RF receive head coil. The MPRAGE sequence was used with 1-mm isotropic resolution, inversion/repetition time = 880/2000 ms, acquisition time: 5 min, FOV: 208 × 256 × 256 matrix, in-plane acceleration factor = 2.

Raw structural MR images were processed using default parameters in CAT12.7 Toolbox (Gaser et al., 2022)^3^ running under standalone MCR version of SPM12 (Wellcome Center for Human Neuroimaging)^4^ massively parallelized on the JURECA High Performance Computing system (Jülich Supercomputing Centre, 2018).

The images were segmented into gray matter, white matter, and cerebrospinal fluid, non-linearly registered and warped to MNI152NLin2009cAsym standard space with 1.5 × 1.5 × 1.5 mm^3^, where a Gaussian filtering (FWHM = 4 mm) was applied.

The CAT12 preprocessing has utilized a unified segmentation (Ashburner and Friston, 2005) to remove B0-inhomogeneities and create an initial segmentation that has been used for (local) intensity scaling and adaptive non-local means denoising (Manjon et al., 2010). An adaptive maximum *a posteriori* (AMAP, Rajapakse et al., 1997) segmentation with a hidden Markov random field (Cuadra et al., 2005) and a partial volume effect model (Tohka et al., 2004) has been adopted to create the final segmentation. Non-linear registration was performed using the Shooting method with modulation (Ashburner and Friston, 2011) as implemented in CAT12.

### 2.4. Statistical analysis

A multiple regression model was applied in CAT12.8 Toolbox (Gaser et al., 2022, see text footnote 3) running in SPM12 (Wellcome Center for Human Neuroimaging, see text footnote 4) and corrected for different brain sizes by applying a global scaling with total intracranial volume.

In order to obtain more comparable results to Takano et al. (2020), we modeled brain volume changes based on T-scores of BMD of the left femoral neck (UKB data field #23300–2.0) for women and men separately, with age as a covariate. A voxel-wise general linear model (GLM) was applied with threshold-free-cluster-enhancement (TFCE with 10,000 permutations) (Smith and Nichols, 2009), accounting for multiple comparisons across the whole brain using an FWE-correction.

Due to possible effects of lifestyle factors and hormonal changes in women, we ran additional sensitivity analyses. The model for women included age, history of hormone replacement therapy (UKB data field #2814-2.0), hysterectomy (i.e., womb removal; #3591-2.0), bilateral oophorectomy (i.e., removal of both ovaries; #2834-2.0), age at menopause (#3581-2.0) and years since the last menstrual period, number of live births (#2734-2.0), frequency of alcohol intake (#1558-2.0), and pack years of smoking (#20161-2.0) as covariates of non-interest. The sensitivity analysis for men included age, pack years of smoking, and alcohol intake frequency as nuisance parameters.

## 3. Results

We report the results with FWE-rate of *p* < 0.01 for voxel-based morphometry (VBM) analysis based on BMD of the left femoral neck for women and men without neurologic or psychiatric impairment (see the sample’s characteristics in Table 1).

**Table 1:** Sample characteristics depicted as mean ± standard deviation and range in the parenthesis.

	Women (*n* = 5,518)	Men (*n* = 7,595)
Age (years)	70.20 ± 3.54 (65.00–82.27)	70.84 ± 3.68 (65.00–82.18)
Age when finished education (years)	16.88 ± 2.55 (5–35)	16.98 ± 2.90 (5–35)
BMI (kg/m^2^)	25.80 ± 4.35 (14.08–55.23)	26.75 ± 3.73 (16.67–49.00)
BMD left femoral neck (T-score)	−1.04 ± 1.00 (−3.98–5.37)	−0.78 ± 1.06 (−4.68–4.40)
Fluid intelligence score	6.43 ± 1.96 (0–13)	6.65 ± 2.07 (0–13)

BMI, body mass index; BMD, bone mineral density.

**Figure 1:**
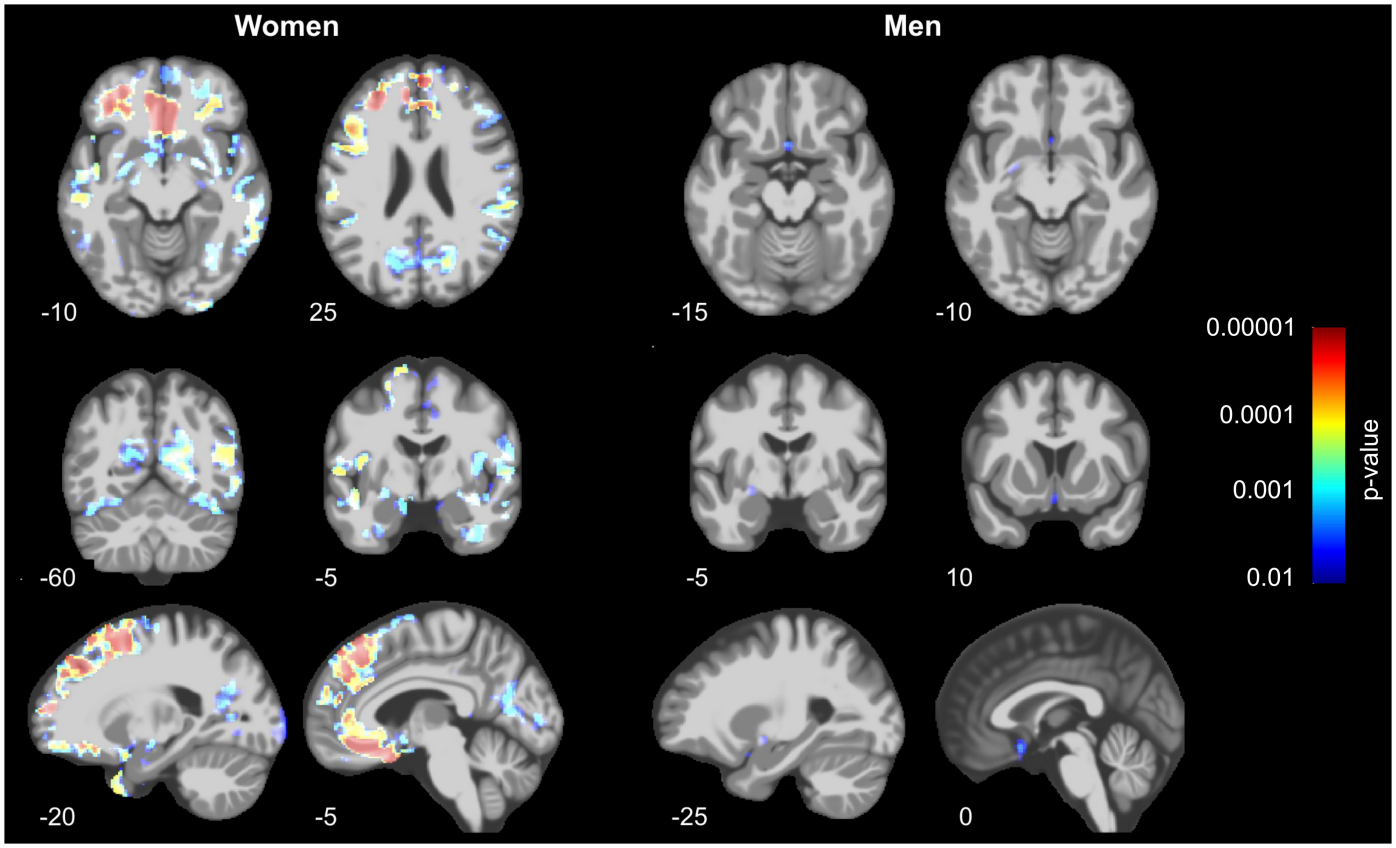
Decrease of gray matter volume in women (left) and men (right) in relation to bone mineral density assessed at the left femoral neck. Prominent results can be seen in women, especially in the prefrontal, parietal and temporo-occipital regions, whereas men show only small effects in a subcallosal area, left basal forebrain and entorhinal area. The results were similar even after controlling for history of hormonal- and lifestyle factors (see Supplementary Figure S1).

As shown in Figure 1, we identified significant TFCE-corrected suprathreshold clusters of decreased GM volume in women with lower BMD of femoral neck in the superior frontal gyri, middle temporal gyri, fusiform gyri, temporal poles, cingulate gyri, precunei, right parahippocampal gyrus and right hippocampus, right ventral diencephalon, and right pre- and postcentral gyrus.

In men, a significant decrease of GMV associated with BMD of the femoral neck was found in subcallosal area, left pallidum and basal forebrain, as well as left entorhinal area.

**Figure 2:**
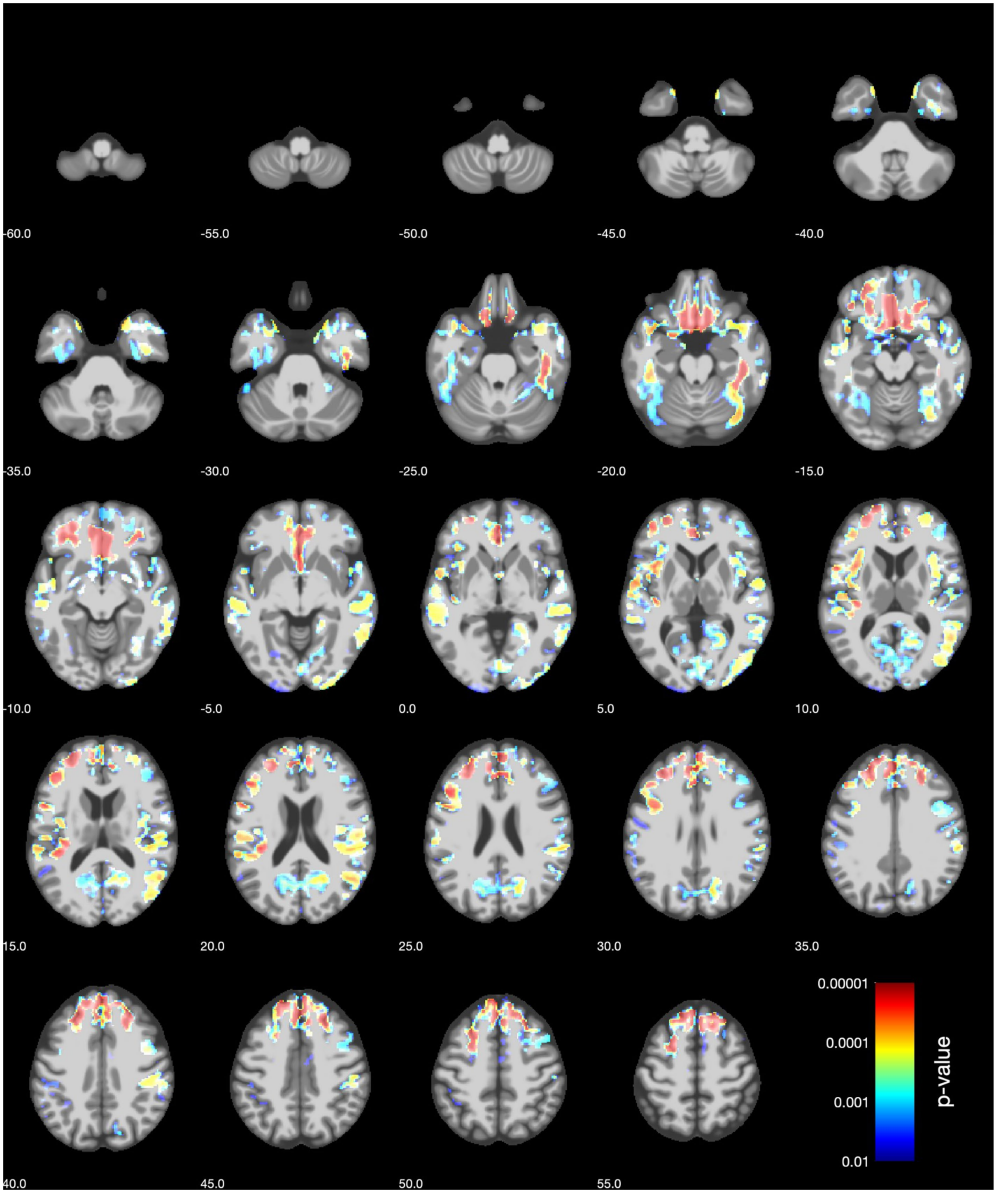
VBM sensitivity analysis results (axial slices) for women for the BMD of the left femoral neck. The covariates included age, history of hormone replacement therapy, hysterectomy, bilateral oophorectomy, age at menopause and years since the last menstrual period, number of live births, frequency of alcohol intake, and pack years of smoking.

The inclusion of covariates related to hormonal- and lifestyle factors did not change the results (Figure 2 and Supplementary Figure S1). See the Supplementary material for an in-depth overview of the results from VBM (sensitivity) analyses.

## 4. Discussion

In the present study, we investigated whether bone mineral density is associated with gray matter volume in healthy older adults of British and Irish descent from the UK Biobank population-based cohort. We found a significant association of decrease in BMD and GM volume in women for the superior frontal gyri, middle frontal and temporal gyri, fusiform gyri, temporal poles, cingulate gyri, precunei, right parahippocampal gyrus and right hippocampus, right ventral diencephalon, and right pre- and postcentral gyrus. Minimal effects could be observed in men in the subcallosal area, left pallidum and basal forebrain, as well as the left entorhinal area.

Previous studies investigating the connection of BMD and brain structure found a decrease of GM volume in hypothalamus, cingulate, parahippocampal gyri, left superior temporal gyrus and left inferior parietal cortex in patients with early AD (Loskutova et al., 2010) and in the left precuneus in male AD patients (Takano et al., 2020). Differences in our results to previous findings from Takano et al. (2020) could stem from several possible factors, namely the sample structure and characteristics (AD patients vs. healthy subjects, calcium supplementation in AD women, sample size, ethnicity). Nevertheless, higher effects in women are expected due to an abrupt hormonal change with onset of menopause, with estrogens being a common denominator of bone remodeling as well as changes in brain structure and function (Kalervo Väänänen and Härkönen, 1996; Morrison et al., 2006; Schatz et al., 2020).

Among the predominantly affected regions were those that are commonly involved in cognitive rather than motor functions (Li et al., 2013; Koyama et al., 2017; Herlin et al., 2021). The region of superior frontal gyri was shown to be affected in aging (Bakkour et al., 2013), but also in the early stages of AD (Apostolova et al., 2008). Furthermore, the superior frontal gyri form a part of the default mode network (Li et al., 2013), which is subjected to alterations in AD, especially in the hippocampal formation and PCC/precuneal region (Karas et al., 2007; Mevel et al., 2011; Yokoi et al., 2018). In the present study, GM volume shrinkage was found in PCC and both precunei only in women, however, a decrease in (para)hippocampal regions was found in both men and women. Our results in men showed a GM volume decrease in the subcallosal area and basal forebrain, which was previously shown to be connected to early stages of AD (Hall et al., 2008; Grothe et al., 2012; Kilimann et al., 2014). However, the effects in men were weaker and potentially affected by possible segmentation issues of blood vessels in those parts.

Osteoporotic patients or subjects with low BMD have an increased risk for developing AD (Chang et al., 2014; Kostev et al., 2018; Zhang et al., 2022; Xiao et al., 2023). Decrease in BMD/a diagnosis of osteoporosis and an abnormal GM volume loss in neurodegenerative diseases share similar risk factors that could explain our results (Friedman et al., 2010; Kostev et al., 2018). Lifestyle factors, such as reduced physical activity, smoking, and alcohol consumption, can result in lower BMD and pose a higher risk for developing dementia (Ward and Klesges, 2001; Livingston et al., 2020; Shojaa et al., 2020). However, our sensitivity analyses demonstrated that a BMD-associated decrease of GM volume in the aforementioned regions remains stable even after controlling for history of hormonal exposure and lifestyle factors. The results could also be explained by secretion of the pituitary hormones; pronounced effects in women can be related to postmenopausal signaling of follicle-stimulating hormone, which is involved in osteoporosis as well as neurodegeneration (Zaidi et al., 2023).

Our study has shown that the effect of low BMD on GM volume decrease can be seen before the potential onset of neurodegenerative diseases. Since we did not screen for participants’ cognitive status, it is possible we can observe the effects of prodromal neurodegeneration. Neurodegenerative diseases were previously shown to affect bone health and increase the risk of fractures, which pose a major threat to personal independence and mortality in older adults (Friedman et al., 2010; Kelly et al., 2020). Although our study design does not allow us to draw any causal conclusions, it adds to the body of literature showing that bone health could affect brain health and *vice-versa*. A possible causal connection of brains influencing the bones has been recently discovered in a Mendelian randomization study (Guo et al., 2023). Further studies that would investigate interrelated longitudinal changes in bone and brain health are necessary to expand our knowledge of the bone-brain crosstalk.

## Data availability statement

Publicly available datasets were analyzed in this study. This data can be found at: https://www.ukbiobank.ac.uk/.

## Ethics statement

The studies involving humans were approved by North West Multi-Centre Research Ethics Committee (MREC). The studies were conducted in accordance with the local legislation and institutional requirements. Written informed consent for participation was not required from the participants or the participants’ legal guardians/next of kin in accordance with the national legislation and institutional requirements.

## Author contributions

RD: Software, Writing – review & editing. FH: Data curation, Resources, Writing – review & editing. CG: Formal analysis, Funding acquisition, Software, Supervision, Writing – review & editing. PK: Conceptualization, Writing – original draft.

## Footnotes

^1^ https://www.ukbiobank.ac.uk/

^2^ https://biobank.ctsu.ox.ac.uk/crystal/crystal/docs/brain_mri.pdf

^3^ https://neuro-jena.github.io/cat/

^4^ https://www.fil.ion.ucl.ac.uk/spm/
