# Supplementary material for: Low bone mineral density is associated with gray matter volume decrease in UK Biobank
Source: Front Aging Neurosci. 2023 Nov 3;15:1287304. doi: 10.3389/fnagi.2023.1287304 (PMC10654785; doi:10.3389/fnagi.2023.1287304)
Supplement: Supplementary file 1 [file Data_Sheet_1.docx]

Supplementary Material

# Supplementary Figures


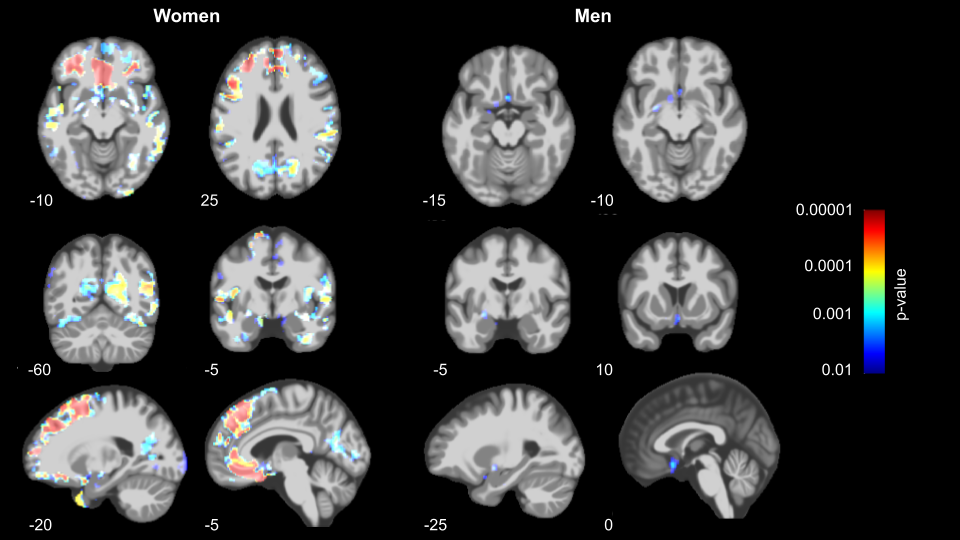


**Figure S1.** VBM sensitivity analysis results for women and men for the BMD of the left femoral neck. The results remain stable even after controlling for history of hormonal exposure and lifestyle factors.


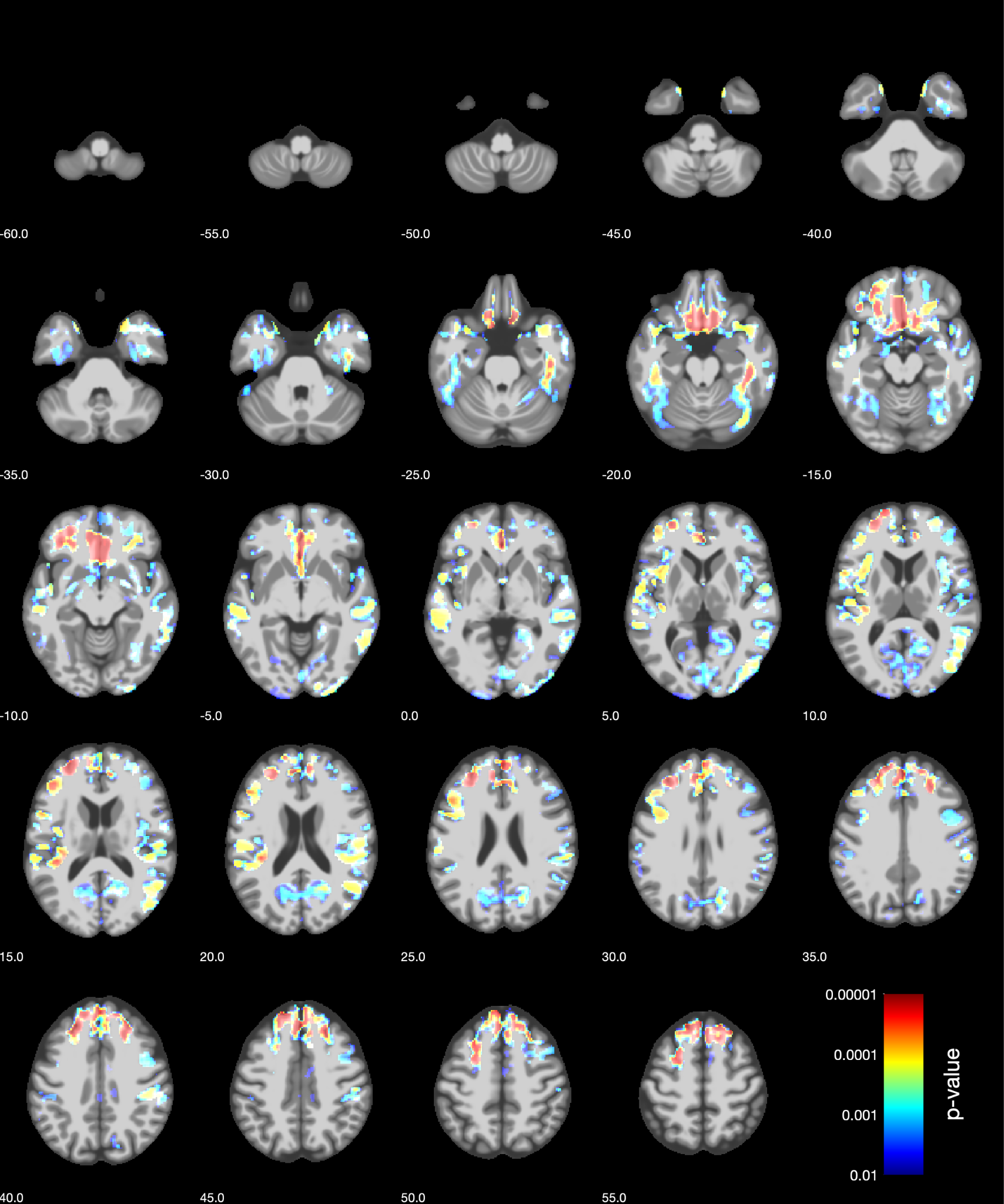


**Figure S2.** VBM analysis results (axial slices) for women for the BMD of the left femoral neck.


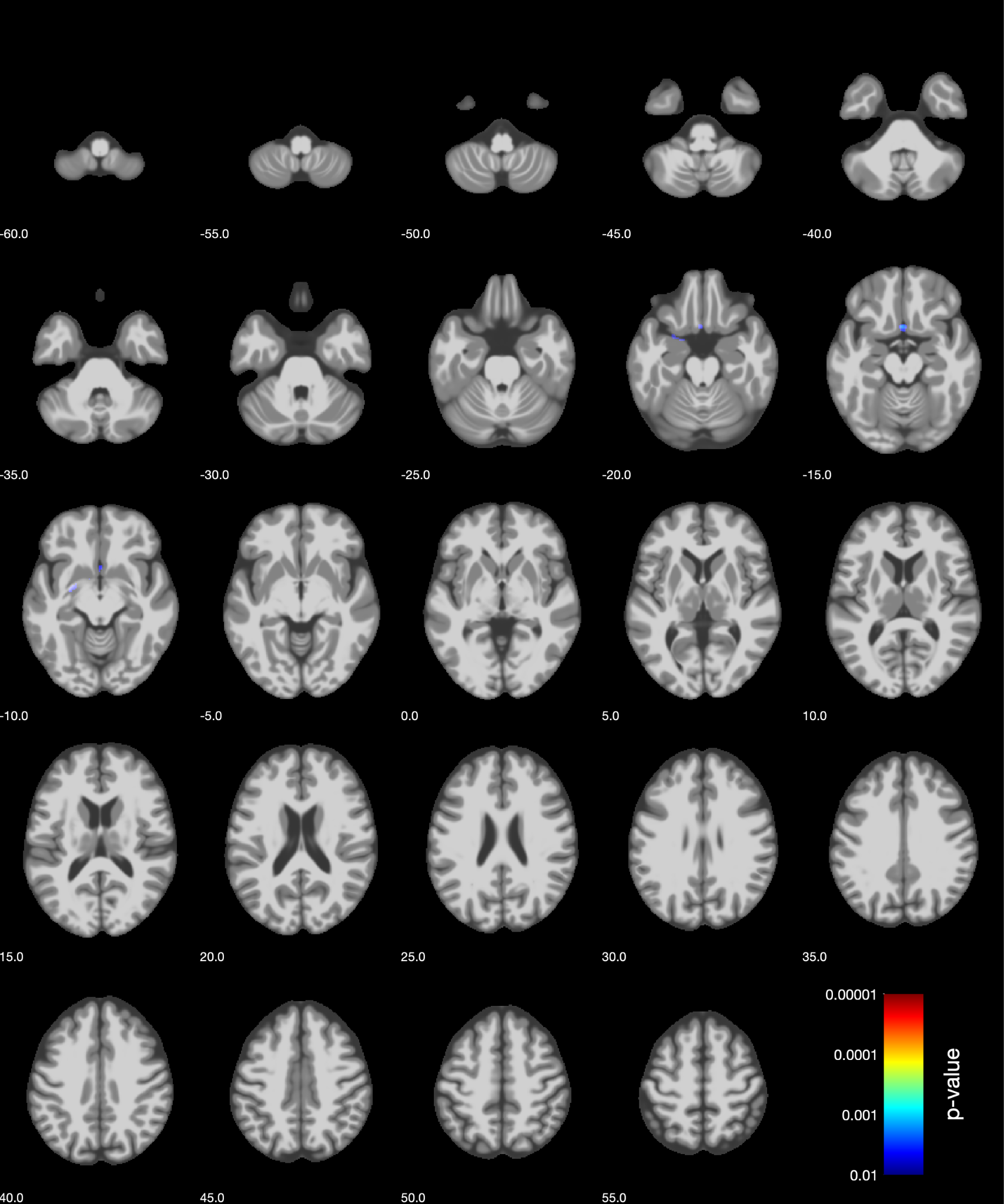


**Figure S3.** VBM analysis results (axial slices) for men for the BMD of the left femoral neck.


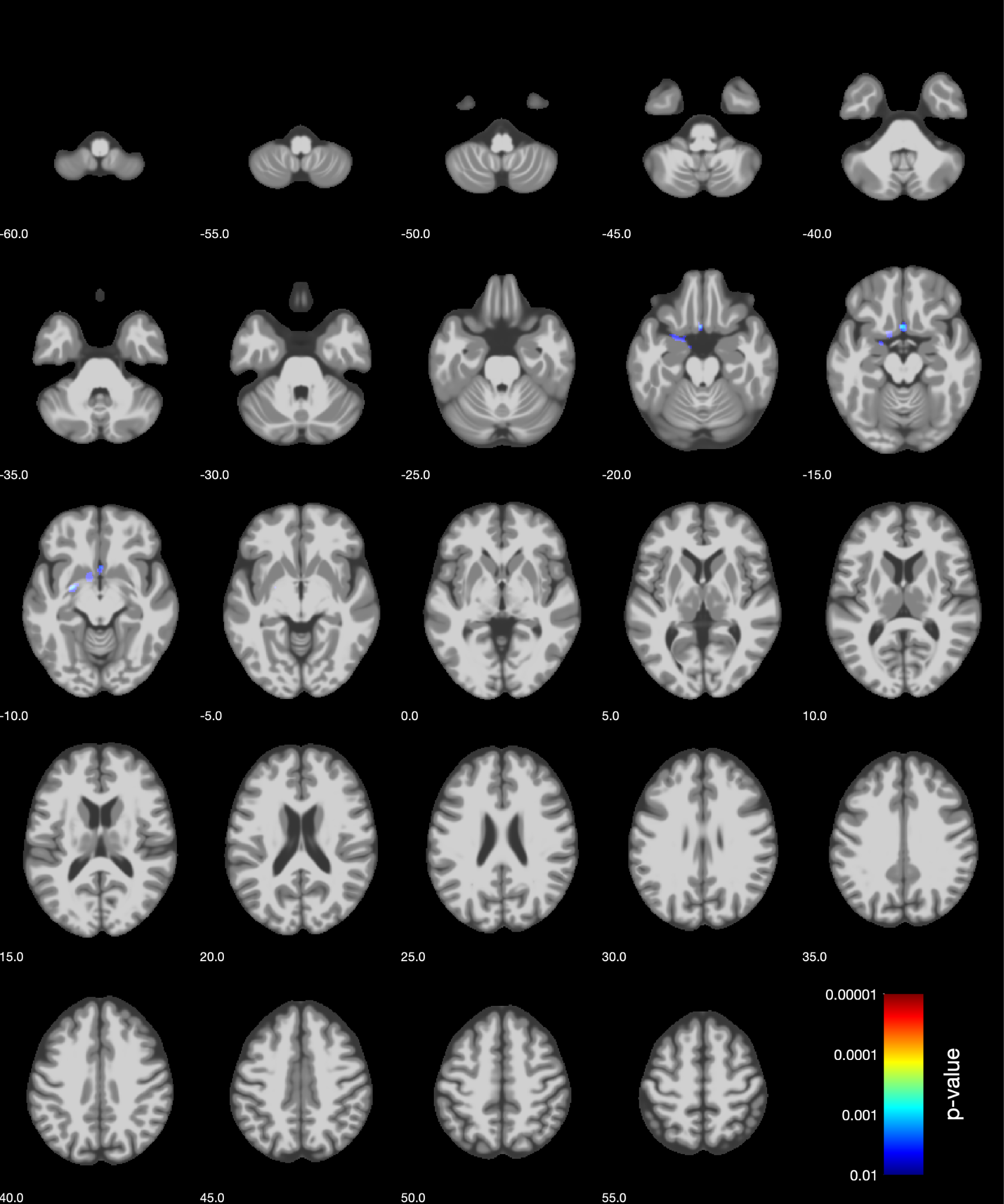


**Figure S4.** VBM sensitivity analysis results (axial slices) for men for the BMD of the left femoral neck. The covariates in the model were age, frequency of alcohol intake, and pack years of smoking.

**
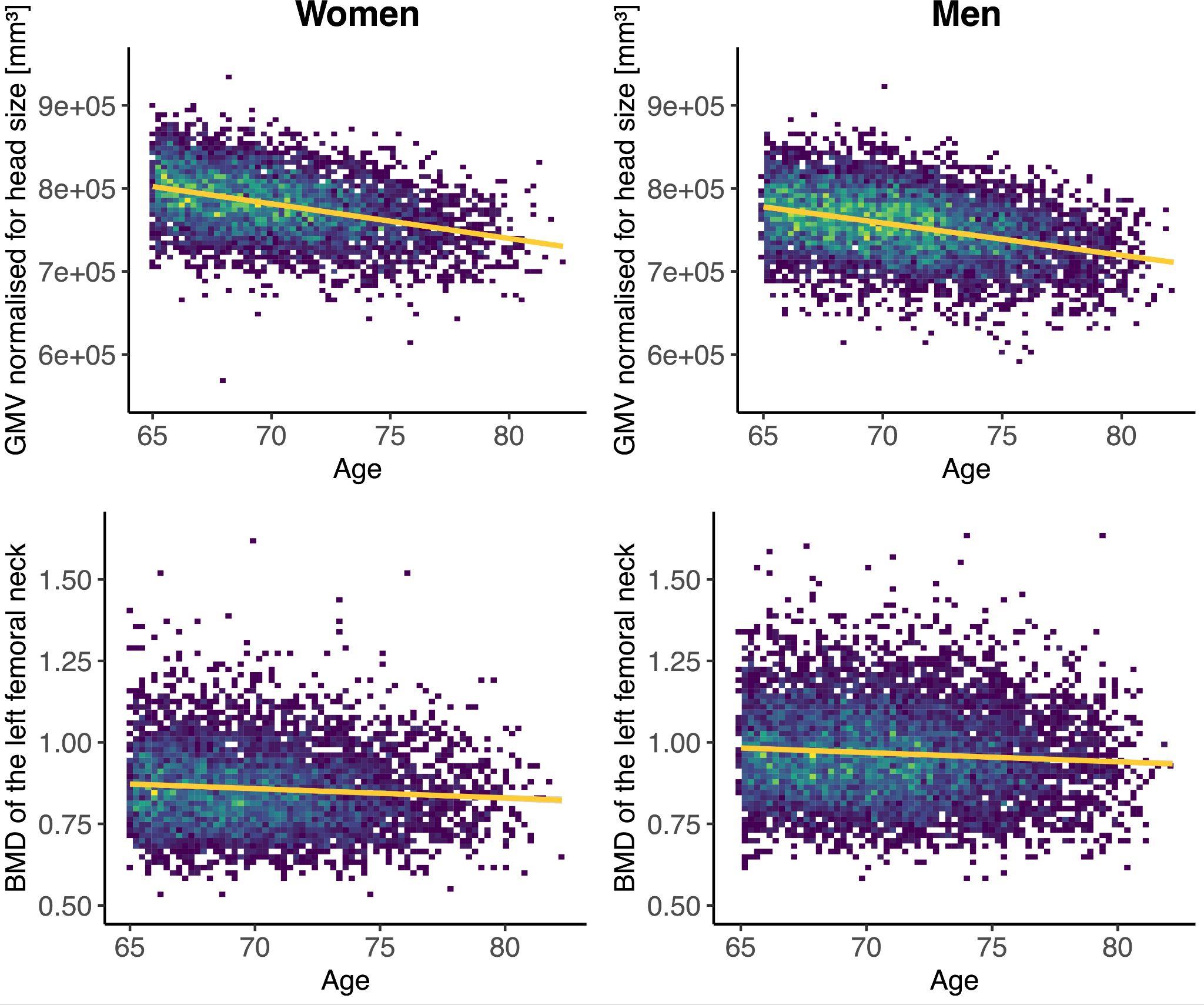
**

**Figure S5.** Grey matter volume (GMV) normalised for head size (UKB #[25005-2.0](http://biobank.ndph.ox.ac.uk/showcase/field.cgi?id=25005)) and raw BMD measures of the left femoral neck (UKB #[23298-2.0](http://biobank.ndph.ox.ac.uk/showcase/field.cgi?id=23298)) in relation to age for women and men. The variance in BMD measure of women in this sample is smaller (s^2^ = 0.99) than in men (s^2^ = 1.11) and the correlation between total GMV in women (*r* = .09, *p* <.01) is higher than in men (*r* = .03, *p* < .01).

**Table 1S.** *VBM analysis results of the left femoral neck T-scores for women*

| *p*-value | Cluster-size | x y z [mm] | Overlap of atlas region |
| --- | --- | --- | --- |

3.5e-07 77814 -2 16 -20 6% L Superior Frontal Gyrus

5% R Superior Frontal Gyrus

4% L Middle Frontal Gyrus

4% R Middle Temporal Gyrus

3% R Superior Frontal Gyrus Medial Segment

3% R Middle Frontal Gyrus

3% L Superior Frontal Gyrus Medial Segment

3% R Fusiform Gyrus

2% L Fusiform Gyrus

2% R Temporal Pole

2% L Anterior Cingulate Gyrus

2% R Precuneus

2% R Inferior Occipital Gyrus

2% R Inferior Temporal Gyrus

2% L Central Operculum

2% R Central Operculum

2% L Temporal Pole

2% L Inferior Temporal Gyrus

2% R Calcarine Cortex

2% L Middle Temporal Gyrus

1% L Precuneus

1% R Cuneus

1% L Superior Temporal Gyrus

1% L Medial Orbital Gyrus

1% R Medial Orbital Gyrus

1% R Lingual Gyrus

1% R Superior Temporal Gyrus

1% R Anterior Cingulate Gyrus

1% R Angular Gyrus

1% R Anterior Insula

1% R Parietal Operculum

1% R Planum Temporale

1% L Planum Temporale

1% R Supplementary Motor Cortex

1% L Precentral Gyrus

1% R Supramarginal Gyrus

1% L Calcarine Cortex

0.0066 34 10 -6 -18 38% R Amygdala

32% R Parahippocampal Gyrus

12% R Ventral DC

9% Background

9% R Hippocampus

0.0079 1 32 -9 -20 100% R Hippocampus

0.0079 1 38 -16 36 100% R Postcentral Gyrus

0.0082 78 0 -28 42 58% L Posterior Cingulate Gyrus

29% R Posterior Cingulate Gyrus

9% L Middle Cingulate Gyrus

4% R Middle Cingulate Gyrus

0.009 91 14 -26 40 43% R Middle Cingulate Gyrus

30% R Posterior Cingulate Gyrus

27% R Precentral Gyrus Medial Segment

|  |
| --- |

**Table 2S.** *VBM analysis results of the left femoral neck T-scores for men*

| *p*-value | Cluster-size | x y z [mm] | Overlap of atlas region |
| --- | --- | --- | --- |

0.0018 109 0 14 -15 71% L Subcallosal Area

29% R Subcallosal Area

0.0045 49 -24 -4 -9 84% L Cerebral White Matter

16% L Pallidum

0.008 29 -20 2 -18 66% L Entorhinal Area

28% L Basal Forebrain

7% L Posterior Orbital Gyrus

0.01 1 -9 3 -10 100% L Basal Forebrain

|  |
| --- |

**Table 3S.** *VBM sensitivity analysis results of the left femoral neck T-scores for women.*

| *p*-value | Cluster-size | x y z [mm] | Overlap of atlas region |
| --- | --- | --- | --- |

1.1e-07 81092 2 16 -18 6% L Superior Frontal Gyrus

5% R Superior Frontal Gyrus

4% L Middle Frontal Gyrus

4% R Middle Temporal Gyrus

3% R Superior Frontal Gyrus Medial Segment

3% R Middle Frontal Gyrus

3% L Superior Frontal Gyrus Medial Segment

3% R Fusiform Gyrus

2% L Fusiform Gyrus

2% R Temporal Pole

2% L Anterior Cingulate Gyrus

2% R Precuneus

2% R Inferior Occipital Gyrus

2% L Central Operculum

2% L Middle Temporal Gyrus

2% L Temporal Pole

2% R Inferior Temporal Gyrus

2% L Superior Temporal Gyrus

2% R Calcarine Cortex

2% R Central Operculum

1% L Inferior Temporal Gyrus

1% L Precuneus

1% R Cuneus

1% L Medial Orbital Gyrus

1% R Medial Orbital Gyrus

1% R Superior Temporal Gyrus

1% R Lingual Gyrus

1% R Anterior Insula

1% R Anterior Cingulate Gyrus

1% R Supramarginal Gyrus

1% R Angular Gyrus

1% L Precentral Gyrus

1% R Parietal Operculum

1% R Planum Temporale

1% L Calcarine Cortex

1% L Planum Temporale

0.0035 506 -21 -100 0 94% L Occipital Pole

4% L Inferior Occipital Gyrus

2% L Calcarine Cortex

0.0056 68 10 -6 -18 37% R Amygdala

24% R Parahippocampal Gyrus

18% R Ventral DC

13% R Hippocampus

7% Background

1% R Entorhinal Area

0.0066 1 15 66 9 100% R Frontal Pole

0.0066 1 38 -16 36 100% R Postcentral Gyrus

0.0092 21 12 -48 -24 67% R Cerebellum White Matter

33% R Cerebellum Exterior

0.0095 6 6 -45 -27 100% R Cerebellum White Matter

0.0099 4 21 -8 -24 100% R Hippocampus

|  |  |  |  |
| --- | --- | --- | --- |

**Table 4S.** *VBM sensitivity analysis results of the left femoral neck T-scores for men*

| *p*-value | Cluster-size | x y z [mm] | Overlap of atlas region |
| --- | --- | --- | --- |

0.00098 177 0 14 -15 67% L Subcallosal Area

32% R Subcallosal Area

1% R Basal Forebrain

0.0023 309 -24 -4 -9 42% L Cerebral White Matter

25% L Basal Forebrain

14% L Entorhinal Area

7% L Pallidum

4% L Accumbens

2% L Posterior Orbital Gyrus

2% L Medial Orbital Gyrus

2% L Subcallosal Area

2% L Amygdala

0.0095 6 -10 -4 -20 50% L Entorhinal Area

33% L Parahippocampal Gyrus

17% L Amygdala

0.0096 4 -27 -15 -27 75% L Cerebral White Matter

25% L Parahippocampal Gyrus

|  |
| --- |

**Medical exclusion criteria:**

1. UKB data-fields #[41202-0.0](http://biobank.ndph.ox.ac.uk/showcase/field.cgi?id=41202) to [41202-0.78](http://biobank.ndph.ox.ac.uk/showcase/field.cgi?id=41202) Diagnoses - main ICD10

Block M80-M85 Bone disease

Block M86-M90 Other osteopathies

C40 Malignant neoplasm of bone and articular cartilage of limbs

C41 Malignant neoplasm of bone and articular cartilage of other and unspecified sites

C79.5 Secondary malignant neoplasm of bone and bone marrow

M89 Other disorders of bone

M90 Osteopathies in diseases classified elsewhere

Block F0-F99 Mental and behavioural disorders

1. UKB data-field #[20003-0.0](http://biobank.ndph.ox.ac.uk/showcase/field.cgi?id=20003) to [20003-3.47](http://biobank.ndph.ox.ac.uk/showcase/field.cgi?id=20003) Treatment/medication code

Use of medications: proton pump inhibitors (nexium, losec, omeprazol), anticonvulsants, corticosteroids, antidepressants, cancer drugs, heparin, warfarin, cyclosporine)

Codes:

1141177532|1140909578|1140865634|1141177526|1140874790|1141145782|1140874816|

1140874976|1140874930|1140868364|1140868426|1140869270|1140858324|1141190580|

1140867876|1140882236|1140867884|1140872072|1140863272|1140872150|1140872160|

1140872216|1140872214|1140872200|1140872198|1140872236|1140872228|1140872152|

1140872160|1140872162|1140872164|1140872228|1140872236|1140872268|1140872280|

1140872284|1140872290|1140872302|1141171932|1141175204|1141175212|1140872172|

1140872098|1140872112|1140872304|1140872306|1141200004|1141200072|141168436|

1140923484|1140927692|2018602634|1140872284|1140881842|1140864956|1140888266|

1140910832|2038459888|2018943436|2018943438|2038459890|1141180320|1141151588|

1140869604|1140869552|1141164182|1140869542|1140869774|1140869772|1140869842|

1140869844|1141173030|1140910876|1140869978|1140910036|1140879600
